# Clinical features and outcomes of gastric neuroendocrine tumors after endoscopic diagnosis and treatment: A digestive endoscopy society of Tawian (DEST): Erratum

**DOI:** 10.1097/MD.0000000000012850

**Published:** 2018-10-12

In the article, “Clinical features and outcomes of gastric neuroendocrine tumors after endoscopic diagnosis and treatment: A Digestive Endoscopy Society of Tawian (DEST)”,^[1]^ which appeared in Volume 97, Issue 38 of *Medicine*, the title should be “Clinical features and outcomes of gastric neuroendocrine tumors after endoscopic diagnosis and treatment: A Digestive Endoscopy Society of Tawian (DEST) multicenter study.”
